# Association between insulin resistance and the development of cardiovascular disease

**DOI:** 10.1186/s12933-018-0762-4

**Published:** 2018-08-31

**Authors:** Valeska Ormazabal, Soumyalekshmi Nair, Omar Elfeky, Claudio Aguayo, Carlos Salomon, Felipe A. Zuñiga

**Affiliations:** 1 Faculty of Biological Sciences, Pharmacology Department, University of Concepcion, Concepción, Chile; 2 Exosome Biology Laboratory, Centre for Clinical Diagnostics, UQ Centre for Clinical Research, Royal Brisbane and Women’s Hospital, Faculty of Medicine + Biomedical Sciences, The University of Queensland, Brisbane, Australia; 3 Faculty of Pharmacy, Department of Clinical Biochemistry and Immunology, University of Concepcion, Concepción, Chile; 4 Department of Obstetrics and Gynecology, Ochsner Baptist Hospital, New Orleans, Louisiana USA

**Keywords:** Insulin resistance, Hyperinsulinemia, Metabolism, Cardiovascular disease, Dyslipidemia

## Abstract

For many years, cardiovascular disease (CVD) has been the leading cause of death around the world. Often associated with CVD are comorbidities such as obesity, abnormal lipid profiles and insulin resistance. Insulin is a key hormone that functions as a regulator of cellular metabolism in many tissues in the human body. Insulin resistance is defined as a decrease in tissue response to insulin stimulation thus insulin resistance is characterized by defects in uptake and oxidation of glucose, a decrease in glycogen synthesis, and, to a lesser extent, the ability to suppress lipid oxidation. Literature widely suggests that free fatty acids are the predominant substrate used in the adult myocardium for ATP production, however, the cardiac metabolic network is highly flexible and can use other substrates, such as glucose, lactate or amino acids. During insulin resistance, several metabolic alterations induce the development of cardiovascular disease. For instance, insulin resistance can induce an imbalance in glucose metabolism that generates chronic hyperglycemia, which in turn triggers oxidative stress and causes an inflammatory response that leads to cell damage. Insulin resistance can also alter systemic lipid metabolism which then leads to the development of dyslipidemia and the well-known lipid triad: (1) high levels of plasma triglycerides, (2) low levels of high-density lipoprotein, and (3) the appearance of small dense low-density lipoproteins. This triad, along with endothelial dysfunction, which can also be induced by aberrant insulin signaling, contribute to atherosclerotic plaque formation. Regarding the systemic consequences associated with insulin resistance and the metabolic cardiac alterations, it can be concluded that insulin resistance in the myocardium generates damage by at least three different mechanisms: (1) signal transduction alteration, (2) impaired regulation of substrate metabolism, and (3) altered delivery of substrates to the myocardium. The aim of this review is to discuss the mechanisms associated with insulin resistance and the development of CVD. New therapies focused on decreasing insulin resistance may contribute to a decrease in both CVD and atherosclerotic plaque generation.

## Background

The pathological processes and risk factors associated with CVD begin as early as during childhood [1]. Notably, obesity associated with an abnormal lipid profile at younger age has been strongly correlated with insulin resistance [2, 3]. As highlighted in the literature, multiple factors such as obesity, abnormal lipid profiles and insulin resistance play key roles in the origin of CVD.

Under physiological conditions, insulin stimulates the use of metabolic substrates in multiple tissues including heart, skeletal muscle, liver, and adipose tissue. In the cardiomyocytes, insulin promotes glucose and fatty acid uptake, but inhibits the use of fatty acids as an energy source. As a result of insulin resistance, the pancreas attempts to compensate by secreting increasing amounts of insulin, resulting in hyperinsulinemia [4].

During insulin resistance and/or hyperinsulinemia, normal glucose tolerance is maintained due a series of physiological changes activated by these phenomenon [5]. Interestingly, a strong correlation between insulin resistance and risk to develop CVD has been established [6]. Several molecular mechanisms contribute to the association between insulin resistance and CVD [4, 7–9]. These mechanisms include the role of insulin resistance in atherosclerosis development, vascular function, hypertension and macrophage accumulation [9].

This review will be focused on the interactions among insulin resistance, and vascular disease, and the molecular mechanism involved. Specifically, the focus will be on the major changes in glucose and lipid metabolism induced by insulin and their impact on the CVD development.

## Insulin signaling

Insulin is a potent anabolic hormone that exerts a variety of effects on many types of cells. Some of the main metabolic actions of insulin are stimulating glucose uptake in skeletal muscles and adipocytes, promoting glycogen synthesis in skeletal muscles, suppressing hepatic glucose production, and inhibiting lipolysis in adipocytes [10]. After ingestion, insulin is secreted from the pancreas and induces the uptake of circulating glucose in its target tissues by binding to an insulin receptor. This binding activates receptor autophosphorylation, which triggers a downstream signaling cascade through the phosphorylation of tyrosine residues of the insulin receptor substrates, IRS (IRS-1 or IRS-2), followed by phosphorylation of phosphatidylinositol 3-kinase (PI3K), phosphoinositide dependent kinase-1, Akt (Akt1 and Akt2), protein kinase C (PKC) and mammalian target of rapamycin (mTOR), as well as ribosomal protein S6 kinase beta 1 (S6K1) [10, 11]. These events result in an increased translocation of the glucose transporter 4 (GLUT4) to the membrane, thus facilitating glucose uptake [12]. After uptake, free glucose is rapidly phosphorylated to glucose 6-phosphate (G6P), which subsequently enters different metabolic pathways [13].

On the other hand, insulin signaling enhances lipid storage in adipocytes by two mechanisms, by stimulating triacylglycerol synthesis and by inhibiting lipolysis. Triglycerides are stored in lipid droplets, which contain lipid droplet proteins, including perilipin [14]. The inhibition of lipolysis occurs through the reduction of cAMP levels and the inhibition of protein kinase A (PKA) activity, hence attenuating HSL (hormone-sensitive lipase) phosphorylation and perilipin, causing a decline in the lipolysis rate [15]. Nutritional needs change during exercise and starvation; triglycerides within the adipocyte lipid droplets are hydrolyzed to fatty acids, acylglycerides and glycerol by activating HSL [16]. In the liver, insulin inhibits glucose production and release, by blocking gluconeogenesis and glycogenolysis through the regulation of expression of phosphoenolpyruvate carboxylase (PEPCK) [17]. Furthermore, insulin can stimulate glycogen synthesis through Akt2 activation, glycogen synthase kinase 3 (GSK3) inhibition, and glycogen synthase (GS) activation via desphosphorylation of serine residues at both the NH_2_ and COOH-terminals of these proteins [18].

**Fig. 1:**
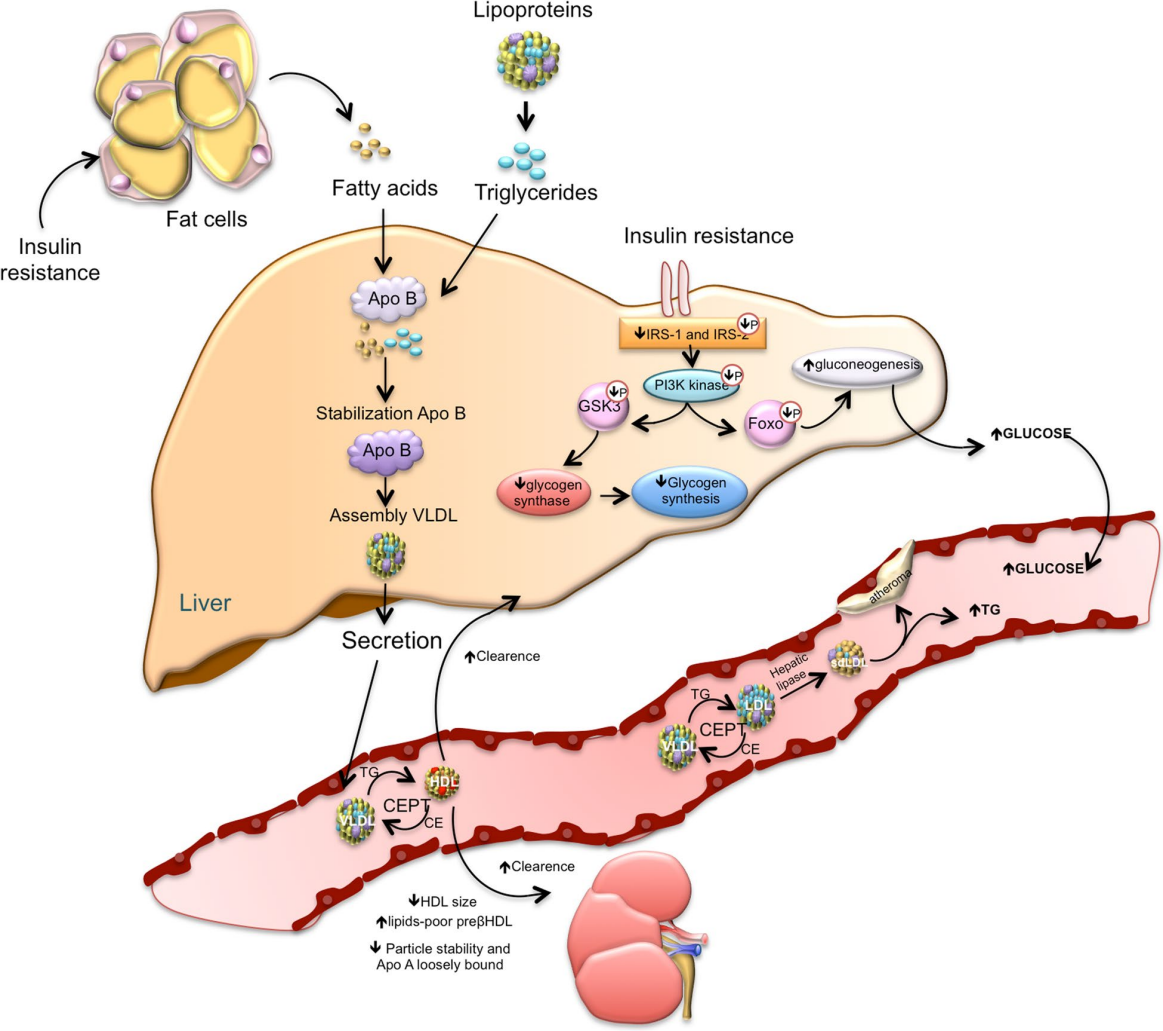
A simplified model of insulin resistance. The loss of suppressive effects of insulin on lipolysis in adipocytes increases free fatty acids. Increased free fatty acids flux to the liver stimulates the assembly and secretion of VLDL resulting in hypertriglyceridemia. Triglycerides (TG) in VLDL are transferred to both HDL and LDL through the action of cholesteryl ester transfer protein (CETP). This process results in a triglyceride-enriched HDL and LDL particle. Triglyceride-enriched HDL is more rapidly cleared from the circulation by the kidney, leaving fewer HDL particles to accept cholesterol from the vasculature. In the glucose metabolism, the insulin resistance results in decreased hepatic glycogen synthesis, owing to decreased activation of glycogen synthase, increased hepatic gluconeogenesis, and glucose delivery by the liver

On the other hand, the vascular actions of insulin are complex, which may have either protective or deleterious effects on the vasculature. The protective effects are related to endothelial nitric oxide synthase (eNOS) activation via PI3K/Akt pathway (Fig. 1). The deleterious effects involve the induction of vascular smooth muscle cell (VSMC) proliferation, vasoconstriction and proinflammatory activity. These vascular effects are mediated through the mitogen-activated protein kinase (MAPK) pathway, which is involved only in the mitogenic effects of insulin, but not in its metabolic effects [19].

## Insulin resistance

Insulin resistance is defined as an experimental or clinical condition in which insulin exerts a biological effect lower than expected. This phenomenon is due to marked defects in the insulin-stimulated glucose uptake, particularly, in glycogen synthesis and, to a lesser extent, glucose oxidation. The effects of insulin resistance in different tissues depend on the physiological as well as metabolic function of the tissues. Due to their high metabolic demand insulin resistance has significant effects on skeletal muscle, adipocytes and liver tissue, which are the main targets of intracellular glucose transport as well as glucose and lipid metabolism [20]. Skeletal muscle and adipocytes accounts for about 60–70% and 10% of insulin-stimulated glucose uptake respectively via the GLUT 4 receptors. Insulin resistance cause impaired glycogen synthesis and protein catabolism in skeletal muscles and inhibit lipoprotein lipase activity in adipocytes leading to an increased release of free fatty acids and inflammatory cytokines such as IL-6, TNFα, and leptin. Additionally, the liver accounts for 30% of insulin-stimulated glucose disposal and insulin resistance leads to impaired glucose output and fatty acid metabolism leading to increased triglyceride content and VLDL secretion from liver [5, 21]. Insulin resistance causes endothelial cell dysfunction by decreasing the production of nitric oxide from endothelial cells and increasing the release of pro-coagulant factors leading to platelet aggregation. In an insulin resistant state, the PI3K pathway is affected whereas the MAP kinase pathway is intact, which causes mitogenic effect of insulin in endothelial cells leading to atherosclerosis [22, 23].

Interestingly, low levels of circulating insulin and insulin resistance have significant physiological roles in regulating metabolic adaptation during starvation and pregnancy. During starvation, low glucose levels leads to decreased secretion of insulin which facilitates the mobilization of glucose from liver, fatty acids and glycerol from adipocytes and amino acids from muscle tissue. These compensatory mechanisms help maintain blood glucose levels and utilization by vital systems like the brain and red blood cells [24]. Insulin resistance is increased in pregnancy particularly from the second to third trimester. This ensures the adequate supply of metabolic substrates and nutrients to the fetus for its proper growth and development [25]. On the contrary, insulin resistance is a key player in the pathogenesis of metabolic diseases like type 2 diabetes [26] and can be observed in several clinical conditions such as breast cancer [27], rheumatoid arthritis [28], polycystic ovary syndrome [29], non-alcoholic fatty liver disease [30], and CVD [31].

The excess of lipids in the cardiomyocyte shunted into non-oxidative pathways results in the accumulation of toxic lipid species (lipotoxicity), which alters cellular signaling and cardiac structure. Disruptions in several cellular signaling pathways such as in mitochondrial dysfunction and endoplasmic reticulum stress have been associated with lipotoxicity. Mediators such as reactive oxygen species (ROS), nitric oxide (NO), ceramide, phosphatidylinositol-3-kinase, diacylglycerol (DAG), ligands of PPAR nuclear receptors, leptin have been proposed to promote these lipotoxic effects and enhances rates of apoptosis [32].

## Cellular mechanisms of insulin resistance

Insulin works on multiple processes, essentially providing an integrated set of signals that allows the correct balance between nutrient supply and demand [33]. In insulin resistance, the target cells fail to respond to ordinary levels of circulating insulin thus higher concentrations of insulin are required for a normal response [34]. In this vein, an insulin resistant state is defined as the impairment of glucose uptake in muscle and an increased gluconeogenesis by the liver resulting in hyperglycemia, both in fasting and postprandial states [35]. A number of theories have been suggested to understand the mechanisms associated with insulin resistance, including genetic defects. Nonetheless, the pathogenesis of insulin resistance can be grouped into: genetic defects, fat derived signal (ectopic lipid accumulation), physical inactivity, obesity, and inflammation [36–38]. One approach to analyze the genetic defect is to define candidate genes based on the present knowledge of the insulin signaling chain. In this regard, some alterations in the genes associated with insulin signaling have been found in insulin resistance and type 2 diabetes. Disruption of IRS-1 and IRS-2 genes in mice showed that IRS-1 knockout mice are insulin resistant but not hyperglycemic [39]. On the other hand, IRS-2-deficient mice are severely hyperglycemic due to abnormalities of peripheral insulin action and failure of β cell secretion [40]. The disruption of Akt1 in mice causes no significant perturbations in metabolism, whereas mice knocked-out for Akt2 show insulin resistance, with a phenotype closely resembling type 2 diabetes of humans [41]. Other mutations that have been identified and studied as possibly responsible for type 2 diabetes are mutations in the insulin receptor, in PI3K, in the liver glucokinase promoter, GLUT4, in the glycogen synthase, and in the protein phosphatase-1. Despite having identified different mutations that may be responsible for the onset of type 2 diabetes, only a few number of individuals are diabetic due to genetic mutations [42]. There may be several other genetic defects, which are not yet identified, that may contribute to the development of insulin resistance or to type 2 diabetes.

In relation to external factors, the increase in free fatty acids (FFA) induced by obesity can trigger insulin resistance through lipid accumulation (ectopic lipids). This may activate atypical PKC that inhibits insulin signaling and insulin-stimulated glucose uptake in skeletal muscles, as well as decreases the insulin-stimulated hepatic glycogen synthesis [43, 44]. This can lead to insulin resistance and increased glucose delivery by the liver [45]. Additionally, FFA triggers insulin resistance by direct activation of Toll-like Receptor 4 (TLR4) and the innate immune response [46].

Furthermore, obesity is associated with inflammatory factors characterized by an increase in the accumulation of ATMs (adipose tissue macrophages). The inflammatory factors increase lipolysis and promote hepatic triglyceride synthesis, and hyperlipidemia due to increased fatty acid esterification. ATM also stimulates inflammatory cytokines that inhibit insulin signaling and expedites hepatic gluconeogenesis, and postprandial hyperglycemia [47, 48].

Other mechanisms that explain insulin resistance are the activation of both mTOR and S6K1 pathways [49]. These activations cause serine phosphorylation of IRS-1, with a subsequent decline in the IRS-1—associated PI3K activity [49]. It has been suggested that under nutrient saturation conditions, S6K1 may negatively regulate insulin signaling and sensitivity [50, 51]. In addition, serine phosphorylation of IRS-1 has been examined under different circumstances. It seems that in addition to the mTOR-S6K1—dependent mechanism, various serine kinases, such as c-Jun NH_2_-terminal kinase (JNK), stress-activated protein kinases, tumor necrosis factor (TNF-α), and PKC, among others, can promote serine phosphorylation of IRS, inducing a decline in insulin signaling strength along the metabolic pathway [49, 52, 53].

## The influence of obesity on insulin resistance

Obesity, in patients with higher body mass index (BMI) levels (≥ 30 kg/m^2^), is associated with a greater risk of cardiovascular disease compared to patients with normal BMI (BMI = 18.8–24.9 kg/m^2^) [54]. Moreover, central obesity is linked to insulin resistance. However, the molecular mechanism by which fat causes insulin resistance is unclear; inflammation due to lipid accumulation, the inhibitory effect of fatty acid oxidation on glucose oxidation, and the secretion of adipocytokines have all been linked to the development of local and systemic insulin resistance [55].

Increasing evidence suggests that the heterogeneity of fat composition and the distribution of adipose tissue can be crucial in the development of insulin resistance and cardiometabolic disruptions [56–58]. Visceral adipose tissue (VAT) has been closely linked to an increasing incidence of insulin resistance [56], T2DM, and a higher risk of cardiovascular disease [59, 60]. VAT is associated with a high production of pro-inflammatory adipocytokines, oxidative stress, and renin–angiotensin–aldosterone system (RAAS) activation [61, 62].

Chronic caloric excess causes increased visceral fat mass due to hypertrophy of individual adipocytes and hyperplasia of adipocyte precursors [63]. As adiposity increases, the adipocytes release chemotactic factors such as monocyte chemoattractant protein-1 (MCP-1), and tumor-necrosis factor-α (TNFα), which modulates an inflammatory response in adipose tissue. MCP-1 initiates the migration of monocytes into VAT and promotes their differentiation into macrophages. Macrophages then secrete large amounts of TNFα, increasing lipolysis and reducing insulin-stimulated glucose transporter 4, triglyceride biosynthesis, and adipocyte storage in the VAT, thus resulting in an increase in circulating triglyceride levels [64]. This event could result in ectopic lipid deposition of toxic fatty acid species (i.e., diacylglycerol, ceramide) in extra-adipose tissue such as the pancreas, kidneys blood vessels, liver, skeletal muscle, and the [65] heart, which leads to an increase of epicardial adipose tissue (EAT) [63].

The increase in EAT leads to cardiac steatosis and to an increase in mass in both ventricles, resulting in ventricular hypertrophy, contractile dysfunction, apoptosis, fibrosis, and impaired left ventricular diastolic function [66–68].

## Insulin resistance and cardiovascular disease

Elevated levels of LDL, smoking, elevated blood pressure and type 1 and type 2 diabetes, are well known risk factors for CVD, however, insulin resistance, hyperglycaemia and inflammation can also lead to and predict adverse cardiovascular events. Furthermore, insulin resistance is related to disorders such as hypertriglyceridemia as well as low levels HDL. Additionally, insulin resistance has been found in approximately 30% of subjects with a diagnosis of hypertension [69]. In 1996, investigators in the Insulin Resistance Atherosclerosis Study (IRAS), showed a direct relation between insulin resistance and atherosclerosis [70] and a follow-up prospective study in a cohort of 2938 patients reported insulin resistance as an important risk factor for CVD [71]. A 2012 meta-analysis of 65 studies, which included 516,325 participants, revealed that insulin resistance, evaluated by HOMA index, was a good predictor for CVD [6]. Using the Archimedes model, and a population representative of young nondiabetic adults aged 20–30 years, the authors came to the conclusion that preventing insulin resistance could avoid approximately 42% of myocardial infarctions in the participants during a simulated follow up period of 60 years [72]. Even though a wealth of studies support the notion that CVD is related to insulin resistance [4, 9, 31, 73–76], there are some controversial reports as well. A study performed by Kozakova et al. reported the association of insulin sensitivity with risk of CVD in young to middle aged men, where as in women, atherosclerosis and plaque formation were independently associated with fasting plasma glucose levels [77]. In addition to insulin resistance, the compensatory hyperinsulinemia associated with insulin resistance can play a critical role in the formation of atherosclerotic plaques by changing the gene expression pattern associated with estrogen receptor, as reported in animal models [78]. Furthermore, hyperglycemia produces alterations in various metabolic and cellular functions [7–9] including dyslipidemia, hypertension, endothelial dysfunction, oxidative stress and alterations in cardiac metabolism. Issues related to the latter alterations are discussed further along in this review.

Approximately 50–70% of required ATP as fuel for the myocardium is produced by (long-chain) fatty acids oxidation. Glycolysis contributes less than 10% of the overall ATP production in the healthy heart [79]. Although there seems to be a preferential use of fatty acids for the production of energy, the heart has the ability to change to another substrate for the generation of ATP, depending on availability, to ensure its energy demand. But also the substrate transporters, GLUT4 (for glucose) and CD36 (for fatty acids), play a role in this dynamic balance of substrate utilization [79]. During injury, the heart shifts from using fatty acids as energetic substrates toward glucose, but this metabolic flexibility is impaired under insulin resistance, leaving to fatty acid as the sole fuel source. This shift induces an increase in the uptake and accumulation of lipid in the heart, producing lipotoxicity [80]. In this sense, the balance between lipid degradation and glucose oxidation could decrease diabetic cardiomyopathy [81]. Likewise, the overexpression of glucose transporter-4 and/or the elimination of CD36 could represent an objective for the development of a new generation drugs for the treatment of diabetic cardiomyopathy.

## Insulin resistance and dyslipidemia

The dyslipidemia induced by insulin resistance and type 2 diabetes (diabetic dyslipidemia) [82] is characterized by the lipid triad: (1) high levels of plasma triglycerides, (2) low levels of HDL, and (3) the appearance of small dense low-density lipoproteins (sdLDL), as well as an excessive postprandial lipemia [35, 82–84]. Hypertriglyceridemia increases the incidence of CVD by 32% in men and 76% in women [85, 86]. A study conducted in 10,038 people with normal blood pressure or pre-hypertension demonstrated dyslipidemia as a strong predictor of development of type 2 diabetes [87]. Frequently, diabetic dyslipidemia precedes type 2 diabetes by several years, suggesting that the abnormal lipid metabolism is an early event in the development of CVD in type 2 diabetes [88].

Obesity is a world-wide epidemic and intimately associated with the development of type 2 diabetes and CVDs. Visceral and epicardial adiposity related to obesity are the major drivers for cardiac disease in these individuals [60]. Obesity has a major effect in modifying the lipoprotein profile and factors associated with systemic and vascular inflammation, and endothelial dysfunction [89]. Abnormal concentrations of lipids and apolipoproteins can produce changes in the production, conversion, or catabolism of lipoprotein particles. These changes may contribute to increased basal lipolysis in obesity and the release of fatty acids into the circulation that consequences a proatherogenic phenotype [19, 90].

## Insulin resistance and lipoproteins profile alterations

VLDL, very low-density lipoprotein, is assembled and produced in the liver, which depends on the availability of substrates and is tightly regulated by insulin [91]. Hepatic VLDL production is induced in the fasting state, which results in increased levels of VLDL in the blood. The increase of lipids from different sources, such as circulating FFA, endocytosis of triglyceride-rich lipoproteins, and de novo lipogenesis, allows for the posttranslational stabilization of apoB and enhances the assembly and secretion of VLDL particles. This leads to VLDL and FFA production, which carries energy between the liver and the adipose tissue [92]. In response to insulin secretion, VLDL synthesis is inhibited to limit the level of plasma triglycerides [83, 93]. Normally, insulin, through PI3K activation, promotes the degradation of apoB, but under insulin resistance this degradation is impaired [92, 94]. Thus, facing a combination of: (1) an excess of fatty acids available, (2) a limited degradation of apoB, and (3) greater stabilization of apoB; an increase in VLDL synthesis is produced, which explains the hypertriglyceridemia observed under insulin resistance [95].

Insulin resistance also decreases lipoprotein lipase activity, a major mediator of VLDL clearance. This effect has a minor contribution in the plasmatic triglycerides level, though it is a mechanism that is also altered. In subjects with type 2 diabetes, hepatic uptake of VLDL, IDL, and LDL is decreased, resulting in increased residence time of these lipoproteins in the plasma [96].

The formation of sdLDL and decreased HDL levels are closely related to insulin resistance. In a prospective study among Atherosclerosis Risk in Communities (ARIC), the plasma levels of sdLDL were associated with risk for incident coronary heart disease (CHD) [97]. Besides, VLDL levels is the major predictor of LDL size [98]. The formation of sdLDL depends on the participation of both, cholesteryl ester transfer protein (CETP) and hepatic lipase. CETP facilitates the transfer of triglycerides from VLDL to LDL and HDL, generating triglyceride-rich LDL and leading to low HDL-C [99]. Triglyceride-rich LDL is a substrate for hepatic lipase, increasing lipolysis of triglyceride-rich LDL, resulting in the formation of sdLDL [100]. Various mechanisms have been suggested to explain the enhanced atherogenic activity of sdLDL, these mechanisms include: (1) lower affinity for the LDL receptor, (2) facilitated entry into the arterial wall, (3) major arterial retention, (4) major susceptibility to oxidation, (5) longer half-time [97]. Increased sdLDL levels represent an increased number of atherogenic particles, which may not be reflected by the levels of LDL, as the sdLDL particles contain less cholesterol (Fig. 1).

The triglyceride enrichment of HDL particles by CETP, combined with the lipolytic action of hepatic lipase, leads to a reduction of plasma HDL-C and apoA-I, which impacts the formation of small dense HDL and leads to an increased catabolism of these particles [100]. A retrospective study conducted in 1932 non-diabetic individuals reported that the ratio of triglyceride to HDL cholesterol ratio can predict insulin resistance and likelihood of metabolic diseases [101]. Additionally, correlation of lipid accumulation products and triglyceride glucose index with insulin resistance and CVD has been demonstrated [102, 103]. Insulin resistance leads to increased release of FFA from adipocytes and the product of fasting plasma FFA by insulin concentration is called adipose tissue insulin resistance. Adipose tissue insulin resistance has been reported as a risk factor for aortic valve calcification, thereby predicting cardiovascular outcomes [104].

## Insulin resistance, hypertension and endothelial dysfunction

Clinical studies have demonstrated that about 50% of hypertensive subjects have comorbid hyperinsulinemia or glucose intolerance, whereas at least 80% of patients with type 2 diabetes have comorbid hypertension [105]. The coexistence of hypertension in diabetic patients greatly enhances the likelihood of these patients developing CVD. It has been suggested that abnormalities in vasodilatation, blood flow, and the renin–angiotensin–aldosterone system (RAAS) can be a linked to hypertension and insulin resistance [105, 106]. An additional cause of hypertension in insulin-resistant patients is over-activity of the sympathetic nervous system, which promotes myocyte hypertrophy, interstitial fibrosis and reduced contractile function, accompanied by increased myocyte apoptosis [107].

In the RAAS, angiotensinogen is converted to angiotensin I by renin, which is then converted to angiotensin II (Ang II) by ACE (angiotensin converting enzyme). Finally, Ang II acts on both AT1 and AT2 receptors. The AT1 receptor mediates all the classic effects of Ang II, such as blood pressure elevation, vasoconstriction, increased cardiac contractility, renal sodium retention, water reabsorption and aldosterone release from by the zona glomerulosa of the adrenal cortex in the adrenal gland [106]. One of aldosterone’s roles is to increase sodium reabsorption in the distal nephron. This effect is to maintain sodium balance via activation of the apical epithelial sodium channel and the basolateral Na^+^, K^+^-ATPase. Aldosterone, however, also exerts effects on the kidney, blood vessels and the myocardium, which can have pathophysiological consequences [108].

**Fig. 2:**
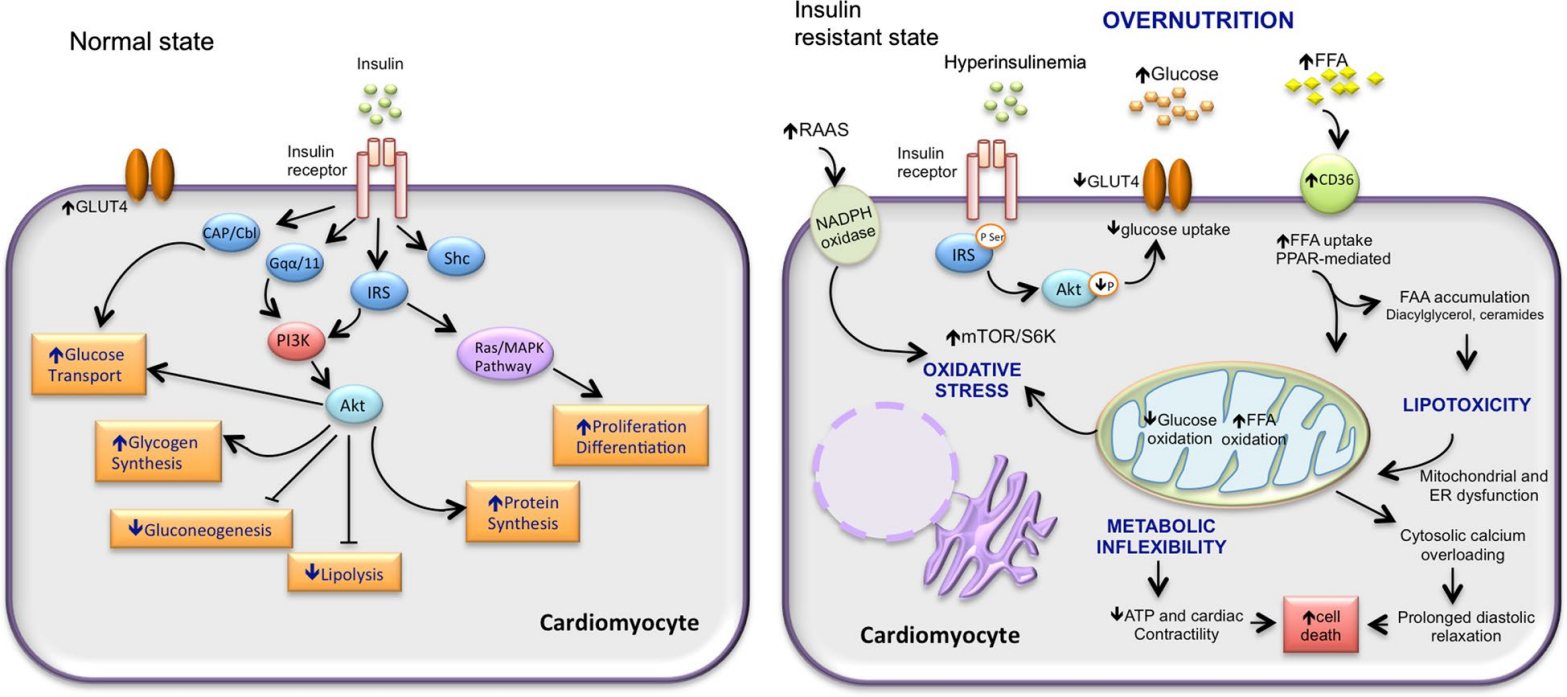
Mechanisms implicated in the development of diabetic cardiomyopathy. Normally, the insulin signaling regulates the glucose and lipids metabolism in heart. Insulin resistance produces a metabolic derangement that results in high lipid oxidation and low of glucose oxidation. The activation of the renin–angiotensin–aldosterone system (RAAS) can cause mitochondrial dysfunction, endoplasmic reticulum stress and oxidative stress. This can results in abnormal Ca^2+^ handling and low ATP production leading to cardiomyocyte death. *ER* endoplasmic reticulum, *FFA* free fatty acids

Literature has shown that hyperglycemia increases transcription of angiotensinogen, ACE and Ang II [105, 109]. On a different matter, an up regulation of RAAS in their cardiovascular system has been found in individuals with type 2 diabetes. An up regulated RAAS may contribute to the development of many diabetic complications, including microvascular and macrovascular diseases [110, 111], in addition, it has been shown that the up regulation of Ang II and the activation of mineralocorticoid receptor by aldosterone might promote insulin resistance through activation of the mTOR–S6K1 signal transduction pathway by inducing phosphorylation in serine residues of IRS [112] (Fig. 2).

Moreover, it has been shown that the activation of RAAS and hyperinsulinemia may synergistically stimulate the MAPK pathway, which exerts an effect damaging to the vascular wall by inducing endothelial dysfunction and promoting atherosclerosis [113]. Additionally, new studies have suggested that the signal transduction pathways of insulin and Ang II share a number of downstream effectors and cross talk at multiple levels [114]. In a related matter, the activation of RAAS (Ang II and aldosterone) and over nutrition contributes to endothelial dysfunction through an increase in the ROS production mediated by nicotinamide adenine dinucleotide phosphate (NADPH)-oxidase, a mechanism that also contributes to hypertension and other CVDs [115]. Indeed ROS leads, in turn, to activation of redox-sensitive kinases such as S6K1 and mTOR, causing an inhibition insulin-PI3K signaling pathway, through phosphorylation at serine residues of IRS-1 [53]. The latter mechanism results in inhibition of downstream signaling of Akt phosphorylation, Glut-4 translocation to the sarcolemma, and Nitric Oxide (NO) production in endothelium [114].

Additionally, hypertension and type 2 diabetes are also associated with a decreased number and impaired function of endothelial progenitor cells, which are circulating bone marrow-derived stem cells that play an important role in the endothelial repair of vascular wall [116]. In some clinical and experimental studies, it has been shown that RAAS inhibition improved insulin signaling and insulin sensitivity [117], however, in others, no beneficial effect has been shown [118]. This discrepancy may be explained by either differences in experimental design or in study populations.

In summary, the activation of TOR/S6K by RAAS, or by over-nutrition, leads to insulin resistance with metabolic and biological consequences. It also leads to impaired myocardial glucose utilization and to a decrease in diastolic relaxation.

## Insulin resistance and endothelial dysfunction

The integrity of the functional endothelium is a fundamental vascular health element. NO is considered to be the most potent endogenous vasodilator in the body, and the reduction in the NO bioavailability is a hallmark of endothelial dysfunction. The endothelial dysfunction contributes to CVD, including hypertension, atherosclerosis and coronary artery disease, which are also caused by insulin resistance [119].

NO participates in vascular wall homeostasis by platelet aggregation, leukocyte adhesion inhibition and anti-inflammatory properties [120]. In physiological conditions, constitutive stimulation of NO production by insulin may play an important role in vascular health maintenance by virtue of its ability to relax vascular smooth muscle. However, in insulin resistance state, the NO synthesis stimulated by insulin is selectively impaired and the compensatory hyperinsulinemia may activate the MAPK pathway, resulting in a vasoconstriction enhancement, inflammation, increased sodium and water retention, resulting in the elevation of blood pressure [113].

In addition, insulin resistance in endothelial cells causes an increased level of prothrombotic factors, proinflammatory markers, and ROS, that lead to an increase in the intracellular levels of adhesion molecule 1 (ICAM-1) and vascular cell adhesion molecule 1 (VCAM-1) [121]. The relation between endothelial function and insulin metabolism is very important. This is because, the association between insulin resistance and endothelial signaling disturbances contributes to inflammation, disrupting the balance between endothelial vasodilator and vasoconstrictor mechanisms and increases cardiovascular risk [10]. A study conducted in non-diabetic patients with suspected myocardial defects reported that insulin resistance measured by HOMA-IR is strongly correlated with endothelial dysfunction with prognostic value [122].

## Chronic hyperglycemia in cardiovascular disease

The increased CVD risk in patients with type 2 diabetes has been known for many years [123]. Patients with diabetes have increased vascular morbidity and mortality, which lowers their life expectancy by approximately 5–15 years. In addition, it has been shown that the CVD incidence is two- to eightfold higher in subjects with type 2 diabetes than in those without diabetes, and this disease accounts for the majority of deaths [124].

To support the latter, epidemiological and pathophysiological studies suggest that hyperglycemia may be largely responsible for CVD. Blood glucose has been reported as an independent predictor of atherosclerosis and blood glucose level greater than 90 mg/dl can lead to atherosclerosis in the carotid artery [125]. Long-term follow up data from patients with type 1 and type 2 diabetes suggest that hyperglycemia is a risk factor for diabetes related diseases and CVDMoreover, it has been suggested by Salvin et al. [126] that a 1 unit increase in the total glycosylated hemoglobin or HbA1C, can increase the risk of CVD by up to 18%. Even in the absence of overt diabetes, impairment in the glucose homeostasis can affect the cardiac autonomic function leading to high risk of cardiac diseases [127].

The detrimental effects of hyperglycemia on cardiomyocytes can be explained by a phenomenon called *hyperglycemic memory*, which is known as a long-term persistence of hyperglycemic stress even after blood glucose normalization [128, 129]. Glucose fluctuations and hyperglycemia trigger inflammatory responses via mitochondrial dysfunction and endoplasmic reticulum stress. This promotes ROS accumulation, which in turn generates cellular damage [130] (Fig. 2). Hyperglycemia may also increase pro-inflammatory and pro-coagulant factors expression, promoting leukocyte adhesion to endothelial cells. It also induces apoptosis and impairs NO release, leading to endothelial dysfunction [7, 131]. For this reason, inflammation leads to insulin resistance and β-cell dysfunction, which further aggravates hyperglycemia, the latter help perpetuate this deregulation. Moreover, changes produced by glucose fluctuations and hyperglycemia can induce long-lasting epigenetic modifications in the promoter of the NF-κB, which appears to be mediated by increased oxidative stress [132].

Another harmful effect of persistent hyperglycemia is the advanced glycation end products (AGEs) generation, which are non-enzymatic glycation products of proteins and lipids as a result of exposure to sugars [133]. In general, the AGEs accumulate in the vessel wall, affecting the structural integrity of the extracellular matrix (ECM) (also known as matrix cell interactions). The latter induces endothelium damage and decreases NO activity. Overall, AGEs contributes to the progression of diabetic complications such as retinopathy, nephropathy and CVD [134].

## Insulin resistance and changes in the cardiac metabolism

The thickest layer of the heart wall is the myocardium, composed of cardiac muscle cells, thus, the knowledge provided by skeletal muscle cell physiology helps explain the cardiac metabolic function [135]. The mammalian heart must contract incessantly; which means the energy requirement for an optimal function is immense and this is an interesting phenomenon because there is no ATP reserve in heart muscle. Instead, energy is stored in cardiac muscle cells in three forms:

1. The first is Phosphocreatine (PCr), which can rapidly donate its high-energy phosphates to produce ATP from ADP [136]. The energy available from PCr is relatively modest, used only during very rapid bursts of exercise [137].
2. The second is glycogen, which forms the endogenous form of energy in the cell. The muscle’s storage capacity for glycogen is limited. However, its advantage is that it consumes much less oxygen compared to fatty acids and is readily available for use as fuel in muscle [138].
3. The third form is triglycerides and FFA. Their oxidation is less efficient compared to glycogen, though it has greater energy input.

It is widely accepted that FFAs are the predominant substrates used in the adult myocardium for ATP production in the mitochondrion [139]. Thus, between 60 and 70% of the energy needed to maintain cardiac work comes from the β-oxidation of FFAs [140]. The levels of circulating FFAs determines largely FFA uptake in the heart [141]. Once the FFA is absorbed, its metabolism is regulated predominantly at the transcriptional level by a family of ligand-activated transcriptional factors namely peroxisome proliferator activator receptor α (PPAR-α) [142].

Depending on their availability or energy requirement (feeding, fasting, and intense exercise), the cardiac metabolic network is highly flexible in using other substrates [143]. The cardiomyocytes are capable of using glucose and lactate that accounts between 25 and 30% and a lesser proportion of amino acids, and ketone bodies [140, 144]. However, glycogen-derived glucose may contribute ≤ 40% of glucose-mediated ATP production, demonstrated in rat heart [145].

Glucose uptake is mediated via glucose transporters. There are two different types of transporters, the Na^2+^-coupled carrier system and the facilitative glucose transporters (GLUT). GLUT1 and GLUT4 are the major players for glucose transport in the heart. GLUT4 represents the major mechanism that regulates glucose entry in the beating heart, with GLUT1 playing a lesser role as it is primarily localized on plasma membranes and is responsible for basal cardiac glucose uptake. GLUT4 is mostly present in the intracellular vesicles at resting stages and is translocated to the plasma membrane upon insulin stimulation [143]. After uptake, free glucose is rapidly phosphorylated to glucose 6-phosphate (G6P), which subsequently enters many metabolic pathways [13]. Glycolysis represents the major pathway in glucose and yields pyruvate for subsequent oxidation. Beside glycolysis, G6P also may be channeled into glycogen synthesis or the pentose phosphate pathway (PPP). The PPP is an important source of NADPH, which plays a critical role in regulating cellular oxidative stress and is required for lipid synthesis [146].

In response to an increased energy demand, heart muscle cells initially rely on carbohydrate oxidation. For example, under stress such as exercise, ischemia and pathological hypertrophy, the substrate preference of glucose can be changed [147]. Under stress, a rapid increase in GLUT4 expression is an early adaptive response that suggests the physiological role of this adaptation is to enhance the replenishment of muscle glycogen stores. When glycogen content is high, the heart preferentially uses glycogen as a source, but when glycogen stores are low, it changes to fatty acid oxidation. This induction can be prevented by a high carbohydrate diet during recovery. The control of metabolism in recovery by glycogen levels underlines its importance as the metabolic muscles reserve [147].

In insulin resistance, the heart is embedded in a rich fatty acid and glucose environment [148–150]. An excess of insulin promotes increased uptake of FFA in the heart due to up regulation of the cluster differentiation protein 36 (CD36) [151], which is a potent FFA transporter; this increases intracellular fatty acids levels and PPAR-α expression. The latter, increases the gene expression in the three stages of fatty acid oxidation by increasing the synthesis of (1) FFA transporters in the cell, (2) proteins that imports FFA to the mitochondrium, and (3) enzymes in the fatty acid oxidation [152]. On the other hand, due to the inhibition of glucose utilization, a glycolytic intermediate accumulates in the cardiomyocytes, which induces glucotoxicity.

Furthermore, when diabetes progresses or when additional stresses are posed on the heart; metabolic mal-adaptation can occur and there is a great loss of metabolic flexibility [153]. The heart decreases its ability to use fatty acids, increasing FFA delivery, and leading to intramyocardial lipid accumulation (ceramides, diacylglycerols, long-chain acyl-CoAs, and acylcarnitines) [154]. This lipid accumulation may contribute to apoptosis, impairing mitochondrial function, cardiac hypertrophy, and contractile dysfunction [155, 156] (Fig. 2). For example, diacylglycerol and fatty acyl-coenzyme (CoA) induce activation of atypical PKC, which results in impaired insulin signal transduction [139]. Ceramides act as key components of lipotoxic signaling pathways linking lipid-induced inflammation with insulin signaling inhibition [157]. On other hand, high lipid contents can induce contractile dysfunction independently of insulin resistance [158]. Therefore, the resultant defect in myocardial energy production impairs myocyte contraction and diastolic function [93, 159] (Fig. 2). These alterations produce functional changes that lead to cardiomyopathy and heart failure [160–163].

In uncontrolled diabetes, the body goes from the fed to the fasted state and the liver switches from carbohydrate or lipid utilization to ketone production in response to low insulin levels and high levels of counter-regulatory hormones [164].

The ketone bodies generated in the liver enter in the blood stream and are used by other organs, such as the brain, kidneys, skeletal muscle, and heart. Disruptions in myocardial fuel metabolism and bioenergetics contribute to cardiovascular disease as the adult heart requires high energy for contractile function [165].

In cardiovascular disease, the capacity of the heart to utilize fatty acids, the heart’s primary fuel, is diminished. In this situation, the heart uses alternative pathways such as ketone bodies as fuel for oxidative ATP production [166]. However, there is still controversy around whether this fuel shift is adaptive or maladaptive. In this sense, recently it has been shown that cyclic ketone bodies preserve “young cardiac phenotype” in old mice [167]. On the other hand, it has been reported that isocaloric ketogenic diet (very low in carbohydrates and high in fats and/or proteins) increases lifespan [168]. The ketogenic diet effect can be mediated by suppressing longevity-related insulin signaling and mTOR pathway, and activation of peroxisome proliferator activated receptor α (PPARα), the master regulator that switches on genes involved in ketogenesis [169].

Several reports suggest that ketogenic diet may be associated with a decreased incidence of risk factors of cardiovascular disease such obesity, diabetes, arterial blood pressure and cholesterol levels, but these effects are usually limited in time [170]. However other reports indicated that cardiac risk factor reductions corresponded with weight loss regardless of a type of diet used [171].

## Others factors that contribute to diabetic cardiomyopathy pathogenesis

Other contributing factors to the diabetic cardiomyopathy pathogenesis are the metabolic abnormalities involving mitochondrial dysfunction, endoplasmic reticulum stress and impaired Ca^2+^ handling [53, 112, 172, 173]. Excessive production of ROS leads to protein, DNA, and membrane damage. In addition, ROS exerts deleterious effects on the endoplasmic reticulum. Oxidative stress and endoplasmic reticulum stress can induce an increase in intracellular Ca^2+^ levels [174]. Excess of Ca^2+^ uptake by the mitochondria leads to Ca^2+^ overload and the opening of the mitochondrial permeability transition pore, which subsequently leads to mitochondrial dysfunction and cell apoptosis. This also contributes to diabetic cardiomyopathy pathogenesis [175, 176].

## Conclusions

Insulin essentially provides an integrated set of signals allowing the balance between nutrient demand and availability. Impaired nutrition contributes to hyperlipidemia and insulin resistance causing hyperglycemia. This condition alters cellular metabolism and intracellular signaling that negatively impact cells. In the cardiomyocyte, this damage can be summarized into three actions: (1) alteration in insulin signaling. (2) Increased substrate accessibility, and (3) inflexibility in metabolism changes. All these effects induce cellular events including: (1) gene expression modifications, (2) hyperglycemia and dyslipidemia, (3) activation of oxidative stress and inflammatory response, (4) endothelial dysfunction, and (5) ectopic lipid accumulation, which, favored by obesity, perpetuates the metabolic deregulation.

Overall, insulin resistance contributes to generate CVD via two independent pathways: (1) atheroma plaque formation and (2) ventricular hypertrophy and diastolic abnormality. Both effects lead to heart failure. Future research is needed to understand the precise mechanism between insulin resistance and its progression to heart failure with a focus on new therapy development.
